# Toxic iron species in lower-risk myelodysplastic syndrome patients: course of disease and effects on outcome

**DOI:** 10.1038/s41375-020-01022-2

**Published:** 2020-09-18

**Authors:** Marlijn Hoeks, Tim Bagguley, Corine van Marrewijk, Alex Smith, David Bowen, Dominic Culligan, Seye Kolade, Argiris Symeonidis, Hege Garelius, Michail Spanoudakis, Saskia Langemeijer, Rian Roelofs, Erwin Wiegerinck, Aurelia Tatic, Sally Killick, Panagiotis Panagiotidis, Oana Stanca, Eva Hellström-Lindberg, Jaroslav Cermak, Melanie van der Klauw, Hanneke Wouters, Marian van Kraaij, Nicole Blijlevens, Dorine W. Swinkels, Theo de Witte, on behalf of the EUMDS Registry Participants, R. Stauder, A. Walder, M. Pfeilstöcker, A. Schoenmetzler-Makrai, S. Burgstaller, J. Thaler, I. Mandac Rogulj, M. Krejci, J. Voglova, P. Rohon, A. Jonasova, J. Cermak, D. Mikulenkova, I. Hochova, P. D. Jensen, M. S. Holm, L. Kjeldsen, I. H. Dufva, H. Vestergaard, D. Re, B. Slama, P. Fenaux, B. Choufi, S. Cheze, D. Klepping, B. Salles, B. de Renzis, L. Willems, D. De Prost, J. Gutnecht, S. Courby, V. Siguret, G. Tertian, L. Pascal, M. Chaury, E. Wattel, A. Guerci, L. Legros, P. Fenaux, R. Itzykson, L. Ades, F. Isnard, L. Sanhes, R. Benramdane, A. Stamatoullas, S. Amé, O. Beyne-Rauzy, E. Gyan, U. Platzbecker, C. Badrakan, U. Germing, M. Lübbert, R. Schlenk, I. Kotsianidis, C. Tsatalas, V. Pappa, A. Galanopoulos, E. Michali, P. Panagiotidis, N. Viniou, A. Katsigiannis, P. Roussou, E. Terpos, A. Kostourou, Z. Kartasis, A. Pouli, K. Palla, V. Briasoulis, E. Hatzimichael, G. Vassilopoulos, A. Symeonidis, A. Kourakli, P. Zikos, A. Anagnostopoulos, M. Kotsopoulou, K. Megalakaki, M. Protopapa, E. Vlachaki, P. Konstantinidou, G. Stemer, A. Nemetz, U. Gotwin, O. Cohen, M. Koren, E. Levy, U. Greenbaum, S. Gino-Moor, M. Price, Y. Ofran, A. Winder, N. Goldshmidt, S. Elias, R. Sabag, I. Hellman, M. Ellis, A. Braester, H. Rosenbaum, S. Berdichevsky, G. Itzhaki, O. Wolaj, S. Yeganeh, O. Katz, K. Filanovsky, N. Dali, M. Mittelman, L. Malcovati, L. Fianchi, A. vd Loosdrecht, V. Matthijssen, A. Herbers, H. Pruijt, N. Aboosy, F. de Vries, G. Velders, E. Jacobs, S. Langemeijer, M. MacKenzie, C. Lensen, P. Kuijper, K. Madry, M. Camara, A. Almeida, G. Vulkan, O. Stanca Ciocan, A. Tatic, A. Savic, C. Pedro, B. Xicoy, P. Leiva, J. Munoz, V. Betes, C. Benavente, M. Lozano, M. Martinez, P. Iniesta, T. Bernal, M. Diez Campelo, D. Tormo, R. Andreu Lapiedra, G. Sanz, E. Hesse Sundin, H. Garelius, C. Karlsson, P. Antunovic, A. Jönsson, L. Brandefors, L. Nilsson, P. Kozlowski, E. Hellstrom-Lindberg, M. Grövdal, K. Larsson, J. Wallvik, F. Lorenz, E. Ejerblad, D. Culligan, C. Craddock, S. Kolade, P. Cahalin, S. Killick, S. Ackroyd, C. Wong, A. Warren, M. Drummond, C. Hall, K. Rothwell, S. Green, S. Ali, D. Bowen, M. Karakantza, M. Dennis, G. Jones, J. Parker, A. Bowen, R. Radia, E. Das-Gupta, P. Vyas, E. Nga, D. Creagh, J. Ashcroft, J. Mills, L. Bond

**Affiliations:** 1 Centre for Clinical Transfusion Research, Sanquin Research, Leiden, The Netherlands; 2 Department of Clinical Epidemiology, Leiden University Medical Center, Leiden, The Netherlands; 3 Department of Hematology, Radboud University Medical Center, Nijmegen, The Netherlands; 4 Epidemiology and Cancer Statistics Group, University of York, York, UK; 5 St. James’s Institute of Oncology, Leeds Teaching Hospitals, Leeds, UK; 6 Department of Hematology, Aberdeen Royal Infirmary, Aberdeen, UK; 7 Department of Hematology, Blackpool Victoria Hospital, Blackpool, Lancashire UK; 8 Department of Medicine, Division of Hematology, University of Patras Medical School, Patras, Greece; 9 Department of Medicine, Sect. of Hematology and Coagulation, Sahlgrenska University Hospital, Göteborg, Sweden; 10 Department of Hematology, Airedale NHS Trust, Airdale, UK; 11 Department of Haematology, Warrington and Halton Teaching Hospitals NHS foundation Trust, Cheshire, UK; 12 Department of Laboratory Medicine, Hepcidinanalysis.com, and Radboudumc Expertise Center for Iron Disorders, Radboud University Medical Center, Nijmegen, The Netherlands; 13 Center of Hematology and Bone Marrow Transplantation, Fundeni Clinical Institute, Bucharest, Romania; 14 Department of Hematology, Royal Bournemouth Hospital, Bournemouth, UK; 15 Department of Haematology, 1st Department of Propedeutic Internal Medicine, National and Kapodistrian University of Athens, Medical School, Laikon General Hospital, Athens, Greece; 16 Department of Hematology, Coltea Clinical Hospital, Bucharest, Romania; 17 Department of Medicine, Division of Hematology, Karolinska Institutet, Stockholm, Sweden; 18 Department of Clinical Hematology, Institute of Hematology and Blood Transfusion, Praha, Czech Republic; 19 Department of Endocrinology, University of Groningen, University Medical Center Groningen, Groningen, The Netherlands; 20 Nijmegen Center for Molecular Life Sciences, Department of Tumor Immunology, Radboud University Medical Center, Nijmegen, The Netherlands; 21 Medical University of Innsbruck, Innsbruck, Austria; 22 Bezirkskrankenhaus, Lienz, Austria; 23 Hanusch Krankenhaus, Vienna, Austria; 24 Klinikum Kreuzschwestern, Wels, Austria; 25 Clinical Hospital Merkur, Zagreb, Croatia; 26 The University Hospital Brno, Brno, Czech Republic; 27 Charles University Faculty of Medicine, Hradec Kralove, Czech Republic; 28 University Hospital, Olomouc, Czech Republic; 29 General University Hospital, 1st Clinic of Internal Medicine, Prague, Czech Republic; 30 General University Hospital, Institute of Hematology and Blood Transfusion, Prague, Czech Republic; 31 University Hospital Motol, Prague, Czech Republic; 32 University Hospital, Aalborg, Denmark; 33 University Hospital, Aarhus, Denmark; 34 University Hospital: Rigshospitalet, Copenhagen, Denmark; 35 Herlev Hospital, Herlev Ringvej, Herlev, Denmark; 36 Odense University Hospital, Odense, Denmark; 37 Hospital Center D’antibes Juan-Les-Pins, Antibes, France; 38 Centre Hospital, Avignon, France; 39 Hospital Avicenne, Bobigny, France; 40 Centre Hospital Boulogne-sur-Mer, Boulogne-sur-Mer, France; 41 Centre Hospital Universitaire Clemenceau, Caen, France; 42 Centre Hospital William Morey, Chalon-sur-Saone, France; 43 Centre Hospital Universitaire, Clermont-Ferrand, France; 44 Hospital Hotel Dieu, Cochin, France; 45 Louis-Mourier Hospital, Colombes, France; 46 CHI Frejus Saint Raphael, Frejus, France; 47 CHU Albert Michallon, Grenoble, France; 48 Hopital Charles-Foix Ap-Hp, Ivry-sur-Seine, France; 49 Hospital Bicetre, Le Kremlin-Bicetre, France; 50 Hospital St Vincent de Paul, Lille, France; 51 CHU Limoges Hospital Dupuytren, Limoges, France; 52 Hospital Edouard Herriot, Lyon, France; 53 CHU Nancy: Hospital Brabois (Vandoeuvre Les Nancy), Nancy, France; 54 CHU de Nice: Hospital l’Archet, Nice, France; 55 Hopital St Louis, Paris, France; 56 Hospital Saint-Antoine, Paris, France; 57 Centre Hospital Marechal Joffre, Perpignan, France; 58 Centre Hospital de Pontoise, Pontoise, France; 59 CHU de Rouen: Hospital Charles-Nicolle, Rouen, France; 60 CHU Hospital Hautepierre de Strasbourg, Strasbourg, France; 61 CHU Toulouse: Hospital Purpan, Toulouse, Toulouse, France; 62 CHRU de Tours, Tours, France; 63 University Hospital Carl Gustav Carus, Dresden, Germany; 64 HELIOS: St. Johannes Hospital in Hamborn, Duisburg, Germany; 65 Heinrich-Heine University Hospital, Dusseldorf, Germany; 66 University Hospital Freiburg, Freiburg, Germany; 67 University Hospital Ulm, Ulm, Germany; 68 Democritus University of Thrace, Alexandroupolis, Greece; 69 General Hospital Attikon, University of Athens Medical School, Athens, Greece; 70 General Hospital G. Gennimatas, Athens, Greece; 71 General Hospital Laikon, University of Athens Medical School, Athens, Greece; 72 General Hospital Sotiria, University of Athens Medical School, Athens, Greece; 73 Hellenic 251 Air Force General Hospital, Athens, Greece; 74 Pammakaristos Hospital, Athens, Greece; 75 Patission Prefectural General Hospital: Halkida, Athens, Greece; 76 St. Savvas Oncology Hospital of Athens, Athens, Greece; 77 General Hospital of Chania, Chania, Greece; 78 University Hospital of Ioannina, Ioannina, Greece; 79 University Hospital of Larissa, Larissa, Greece; 80 General University Hospital of Patras, Patras, Greece; 81 St. Andreas General Hospital, Patras, Greece; 82 General Hospital of Thessaloniki George Papanikolaou, Pilea Chortiatis, Greece; 83 Metaxa Hospital, Piraeus, Greece; 84 General Hospital of Serres, Serres, Greece; 85 Hippokration—General Hospital of Thessaloniki, Thessaloniki, Greece; 86 Theageneio General Hospital, Thessaloniki, Greece; 87 HaEmek Medical Center, Afula, Israel; 88 Barzilai Medical Center, Ashkelon, Israel; 89 Asaf-Harofe Medical Center, Be’er Ya’akov, Israel; 90 Soroka Medical Center, Beersheba, Israel; 91 Bnai Zion Medical Center, Haifa, Israel; 92 Carmel Medical Center, Haifa, Israel; 93 Rambam Medical Centre, Haifa, Israel; 94 Wolfson Medical Center, Holon, Israel; 95 Hadassah Medical Center, Jerusalem, Israel; 96 Meir Medical Center, Kfar Saba, Israel; 97 The Western Galilee Hospital, Nahariya, Israel; 98 Nazareth Towers Medical Center, Nazareth, Israel; 99 Laniado Hospital, Netanya, Israel; 100 Rabin Medical Center, Petah Tikva, Israel; 101 Baruch Padeh Medical Center Poriya, Tiberias, Israel; 102 Kaplan Medical Center, Rehovot, Israel; 103 Ziv Medical Center, Safed, Israel; 104 Tel Aviv Sourasky Medical Centre, Tel Aviv, Israel; 105 IRCCS San Matteo Hospital Foundation, Pavia, Italy; 106 University Cattolica del Sacro Cuore, Policlinico Gemelli, Rome, Italy; 107 VU University Medical Center, Amsterdam, The Netherlands; 108 Rijnstate Hospital, Arnhem, The Netherlands; 109 Jeroen Bosch Hospital, Den Bosch, The Netherlands; 110 Slingeland Hospital, Doetinchem, The Netherlands; 111 Gelderse Vallei Hospital, Ede, The Netherlands; 112 Elkerliek Hospital, Helmond, The Netherlands; 113 Radboudumc, Nijmegen, The Netherlands; 114 Bernhoven Hospital, Uden, The Netherlands; 115 Maxima Medical Center, Veldhoven, The Netherlands; 116 Warszawski Uniwersytet Medyczny, Warsaw, Poland; 117 Centro Hospitalar de Lisboa, Lisbon, Portugal; 118 Districtual Hospital, Brasov, Romania; 119 Coltea Clinical Hospital, Bucharest, Romania; 120 Fundeni Clinical Institute, Bucharest, Romania; 121 Clinical Center of Vojvodina, Novi Sad, Serbia; 122 Hospital del Mar, Barcelona, Spain; 123 Hospital Universitari Germans Trias i Pujol, Barcelona, Spain; 124 Hospital Del Sas, Jerez De La Frontera, Cadiz, Spain; 125 Hospital Universitario Puerta del Mar, Cadiz, Spain; 126 Institute de Investigacion Biomedica, Lleida, Spain; 127 Hospital Clinico Universitario San Carlos, Madrid, Spain; 128 Hospital Universitario Meseguer, Murcia, Spain; 129 Hospital Universitario Virgen de la Arrixaca, Murcia, Spain; 130 Hospital Universitario Central de Asturias, Oviedo, Spain; 131 Hospital Universitario de Salamanca, Salamaca, Spain; 132 Hospital Clinico Universitario de Valencia, Valencia, Spain; 133 Hospital Dr. Peset, Valencia, Spain; 134 Hospital Universitario La Fe, Valencia, Spain; 135 Malarsjukhuset, Eskilstuna, Sweden; 136 Sahlgrenska University Hospital, Göteborg, Sweden; 137 Teaching Hospital of Halmstad, Halmstad, Sweden; 138 University Hospital Linköping, Linköping, Sweden; 139 Sunderby Hospital, Lulea, Sweden; 140 Lund University Hospital, Lund, Sweden; 141 Orebro University Hospital, Orebro, Sweden; 142 Karolinska University Hospital, Stockholm, Sweden; 143 Södersjukhuset, Stockholm, Sweden; 144 Sundsvalls sjukhus, Sundsvall, Sweden; 145 Umea Regional Hospital, Umea, Sweden; 146 Uppsala University, Uppsala, Sweden; 147 Aberdeen Royal Infirmary, Aberdeen, UK; 148 Queen Elizabeth Hospital, Birmingham, UK; 149 Blackpool Victoria Hospital, Blackpool, UK; 150 Royal Bournemouth Hospital, Bournemouth, UK; 151 Bradford Royal Infirmary, Bradford, UK; 152 Addenbrooke’s Hospital, Cambridge, UK; 153 Western Infirmary, Glasgow, UK; 154 Harrogate District Hospital, Harrogate, UK; 155 Huddersfield Royal Infirmary, Huddersfield, UK; 156 Hull and East Yorkshire Hospitals NHS Trust, Hull, UK; 157 Leeds Teaching Hospitals, Leeds, UK; 158 Christie Hospital, Manchester, UK; 159 Royal Victoria Infirmary, Newcastle upon Tyne, UK; 160 Northampton General Hospital, Northampton, UK; 161 City Hospital, Nottingham, UK; 162 John Radcliffe Hospitals NHS Trust, Oxford, UK; 163 Airedale NHS Trust, Steeton, UK; 164 Royal Cornwall Hospital, Truro, UK; 165 Mid Yorkshire Hospitals, Wakefield, UK; 166 Worcestershire Acute Hospitals NHS Trust, Worcester, UK; 167 York Hospital, York, UK

**Keywords:** Myelodysplastic syndrome, Myelodysplastic syndrome

## Introduction

Red blood cell transfusions (RBCT) remain the cornerstone of supportive care in lower-risk myelodysplastic syndrome (LRMDS) [1]. Transfusion dependency in LRMDS patients is associated with inferior outcomes, mainly attributed to severe bone marrow failure [2]. However, iron toxicity, due to frequent RBCT or ineffective erythropoiesis, may be an additional negative prognostic factor [3–6]. Recently, much progress has been made in unraveling the iron metabolism. The peptide hormone hepcidin is the key regulator by inhibiting iron uptake through degradation of ferroportin, a cellular iron exporter [7]. Erythroferrone and GDF15, produced by erythroblasts, inhibit hepcidin production, which leads to increased uptake and cellular release of iron for the purpose of erythropoiesis [8].

The pathophysiology of iron metabolism in MDS is still not completely understood. Exceedingly high reactive oxygen species (ROS) levels are associated with iron toxicity, disease development, and progression in MDS patients [9–12]. Malondialdehyde (MDA), resulting from lipid peroxidation of polyunsaturated fatty acids, is a biomarker of oxidative stress [10, 12]. Currently, little is known about the prognostic impact of ROS in MDS patients.

The aim of this study is twofold: (1) describe iron and oxidative stress parameters over time in LRMDS patients and (2) to assess their effect on overall and progression-free survival.

## Materials and methods

The EUMDS registry prospectively collects observational data on newly diagnosed LRMDS patients from 148 centers in 16 countries in Europe and Israel as of January 2008. All patients provided informed consent. Clinical data were collected at baseline and at each six-monthly follow-up visit. Serum samples were collected prospectively at each visit from 256 patients included in six participating countries. Conventional iron parameters were measured with routine assays. We additionally analyzed hepcidin, growth differentiation factor 15 (GDF15), soluble transferrin receptor (sTfR), non-transferrin bound iron (NTBI), labile plasma iron (LPI), and MDA. Subjects were prospectively followed until death, loss to follow-up, or withdrawal of consent.

All iron parameters were measured centrally at the department of Laboratory Medicine of the Radboudumc, Nijmegen, The Netherlands. Serum samples were collected just prior to transfusion in transfusion-dependent patients and stored at −80 °C. Details on the assays and reference ranges of hepcidin, GDF15, sTfR, NTBI, LPI, and MDA are provided in the supplement.

The Spearman rank test was used to evaluate correlations between iron parameters. We stratified the results by transfusion dependency per visit and the presence of ring sideroblasts. When evaluating temporal changes in iron parameters, with linear quantile mixed models, we excluded patients from the timepoint they received iron chelation therapy. Overall survival (OS) was defined as the time from MDS diagnosis to death or, in case of progression-free survival, to date of progression or death; patients still alive at the end of follow-up were censored. Time-dependent Kaplan–Meier curves and cox proportional hazards models were used.

## Results

In total, 256 consecutive patients, were included in this study. Over five six-monthly visits, 1040 samples were collected. Table 1 describes the patient characteristics. Most patients without ring sideroblasts were transfusion-independent at diagnosis (nonRS-TI; 55.9%), 18.8% with ring sideroblasts were transfusion-independent (RS-TI), 18.4% without ring sideroblasts were transfusion-dependent (nonRS-TD), and 7% with ring sideroblasts were transfusion-dependent patients (RS-TD). The median follow-up time was 6.6 years (95% CI 5.9–7.0).

**Table 1:** Baseline characteristics.

	*N* (%)
Total	256 (100.0)
Sex	
Males	169 (66.0)
Females	87 (34.0)
Age	
35–44	2 (0.8)
45–54	7 (2.7)
55–64	51 (19.9)
65–74	78 (30.5)
75+	118 (46.1)
Mean (sd)	72.1 (9.5)
Median (min–max)	74.0 (37.0–95.0)
MDS diagnosis	
RCMD	114 (44.5)
RARS	56 (21.9)
RA	45 (17.6)
RAEB-1	16 (6.3)
RCMD-RS	10 (3.9)
5q-syndrome	10 (3.9)
MDS-U	5 (2.0)
Group	
NonRS-TI	143 (55.9)
NonRS-TD	47 (18.4)
RS-TI	48 (18.8)
RS-TD	18 (7.0)
IPSS-R category	
Very low/low	195 (76.2)
Intermediate	23 (9.0)
High/very high	4 (1.6)
Not known	34 (13.3)
IPSS category	
Low risk	144 (56.3)
Intermed-1	75 (29.3)
Intermed-2	1 (0.4)
Not known	36 (14.1)
Karnofsky performance status	
Able to work and normal activity	193 (75.4)
Unable to work	48 (18.8)
Unable to care for self	1 (0.4)
Not known	14 (5.5)
Comorbidity index	
Low risk	158 (61.7)
Intermediate risk	79 (30.9)
High risk	19 (7.4)
EQ5D index score	
Mean (sd)	0.77 (0.24)
Median (p10–p90)	0.80 (0.52–1.00)
ESA	
No	159 (62.1)
Yes	97 (37.9)
Iron chelation	
No	241 (94.1)
Yes	15 (5.9)
Desferoxamine	5 (2.0)
Deferiprone/deferasirox	11 (4.3)
Hypomethylating agents	
No	245 (95.7)
Yes	11 (4.3)
Overall survival	
Median (95% CI)	4.8 (3.9—not reached)
Cause of death	
MDS unrelated	15 (34.1)
MDS related	24 (54.5)
Unknown	5 (11.4)
Follow-up time (censored last EUMDS visit)	
Median (95% CI)	6.6 (5.9–7.0)

*sd* standard deviation, *MDS* myelodysplastic syndrome, *RCMD* refractory cytopenia with multilineage dysplasia, *RARS* refractory anemia with ring sideroblasts, *RA* refractory anemia, *RAEB* refractory anemia with excess blasts, *RCMD-RS* refractory cytopenia with multilineage dysplasia with ring sideroblasts, *MDS-U* myelodysplastic syndrome unspecified, *RS* ring sideroblasts, *TI* transfusion-independent, *TD* transfusion-dependent, *IPSS(-R)* (revised) international prognostic scoring system, *EQ5D* EuroQoL five dimension scale, *ESA* erythroid stimulating agents.

LPI was positively correlated with transferrin saturation (TSAT) (*r* = 0.15, *p* < 0.001, Fig. S1). LPI values increased exponentially at TSAT values above 80%. This effect was most pronounced in the transfusion-dependent groups, but also observed in the RS-TI group. MDA was weakly correlated with NTBI (*r* = 0.09, *p* = 0.069) and negatively correlated with hemoglobin level (*r* = −0.1, *p* = 0.033). GDF15 and hepcidin were negatively correlated in the RS-TI and nonRS-TD group and significantly negatively correlated in the RS-TD group (*r* = −0.34, *p* = 0.007, Fig. S2).

Serum ferritin levels were elevated in all subgroups with a mean value of 858 µg/L at visit 5. The highest serum ferritin levels were observed in the RS-TD group (mean value at visit 5: 2092 µg/L, Table S1). Serum ferritin increased significantly per visit in the RS-TD group (beta 454.46 µg/L; 95% CI 334.65–574.27), but not in the other groups (Table S2).

All subgroups, except for the nonRS-TI, had elevated TSAT levels. TSAT levels were most markedly increased in the RS-TD group with a mean TSAT of 88% at visit 5 (Table S1). In both transfusion-dependent groups the median increase per visit was significant (Table S2).

LPI was elevated in the RS-TD group exclusively with a mean value of 0.59 µmol/L at visit 5 (Table S1). NTBI was elevated in all subgroups, with the highest values in the RS-TD group (Table S1). The increase in median NTBI level was significant in both transfusion-dependent groups (Table S2).

Hepcidin levels were markedly elevated in the nonRS-TD group. Interestingly, hepcidin levels were lower in the RS-TD group, probably reflecting ineffective erythropoiesis, likewise supported by lower hepcidin/ferritin ratios in RS groups (Table S1). Median hepcidin levels increased over time in the transfusion-dependent subgroups only (Table S2).

GDF15 levels, analyzed in the light of its potential role in hepcidin suppression, were increased in all subgroups (Table S1). The RS subgroups had higher GDF15 levels compared to the nonRS groups, reflecting increased erythropoiesis.

Mean sTfR levels were within the reference range in all subgroups except for the RS-TI group, which showed elevated levels, reflecting increased erythropoiesis (Table S1).

MDA levels were within the reference range in the nonRS-TI group and above the upper limit of the reference range in all other subgroups with the highest levels in the RS-TD group (Table S1). MDA levels at diagnosis were markedly higher in the RCMD-RS group compared to other subtypes (Table S3.1). As expected, in the group with elevated MDA levels, the transfusion density was markedly higher as compared with patients with low MDA levels (Table S3.2). Overall MDA levels increased over time (*p* < 0.0001). The steepest increase was observed in transfusion-dependent patients, with the highest median levels over time in the RS-TD group (Table S3.3).

### Overall survival (OS)

**Fig. 1:**
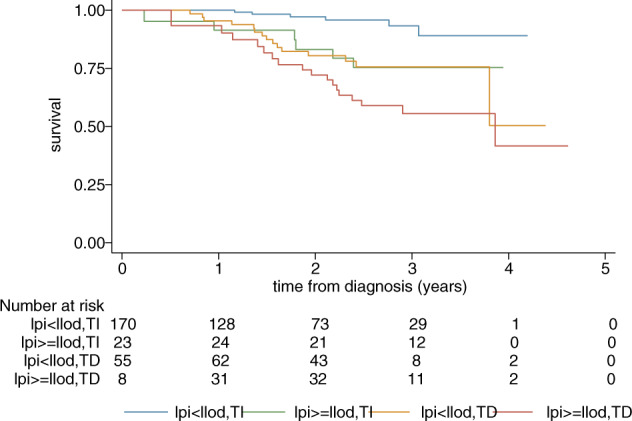
Kaplan–Meier curve overall survival stratified by labile plasma iron above or below the lower limit of detection and transfusion status as time-dependent variables. LPI labile plasma iron, LLOD lower limit of detection, TI transfusion-independent, TD transfusion-dependent.

Figure 1 shows a Kaplan–Meier curve for OS, stratified by LPI above or below the lower limit of detection (LLOD) and transfusion status as time-varying variables. Transfusion-dependent patients with elevated LPI levels have inferior OS compared to other subgroups. The Cox model shows an adjusted hazard ratio (HR) for OS, corrected for age at diagnosis and IPSS-R, of 2.7 (95% CI 1.5–5.0, *p* = 0.001) for LPI > LLOD. With the transfusion-independent group with LPI values <LLOD as a reference, the HR for OS in the transfusion-independent group with LPI > LLOD was 4.5 (95% CI 1.4–13.9, *p* = 0.01), for the transfusion-dependent group with LPI < LLOD: 3.9 (95% CI 1.5–10.4, *p* = 0.006), and for the transfusion-dependent group with LPI > LLOD: 6.7 (95% CI 2.5–17.6, *p* < 0.001, Table S4).

The adjusted HR for OS for elevated NTBI was 1.6 (95% CI 0.8–3.1, *p* = 0.17). Transfusion-independent patients with normal NTBI levels have superior OS when compared with the other subgroups, who have significantly increased HRs for OS (Table S5).

Elevated TSAT (>80%) alone did not influence OS. However, when we repeated the analysis in the whole EUMDS registry as a dichotomous and continuous variable (*n* = 1076, 2853 visits), elevated TSAT did influence OS with an adjusted HR of 2.1 (95% CI 1.6–2.7, *p* < 0.001) and 1.009 (95% CI 1.004–1.014, *p* < 0.001), respectively. Transfusion-dependent patients with a TSAT ≥ 80% had the worst OS with an adjusted HR of 4.2 (95% CI 2.9–5.9, *p* < 0.001).

### Progression-free survival

In line with the effect of LPI on OS progression-free survival is significantly inferior in transfusion-dependent patients with LPI levels >LLOD (HR 9.2, 95% CI 3.8–22.5, *p* < 0.001).

## Discussion

The results of this study suggest that LRMDS patients who are transfusion-dependent and have a MDS subtype with ring sideroblasts have the highest levels for markers that reflect iron toxicity. Likewise, the highest hepcidin levels were observed in the transfusion-dependent nonRS group, but importantly, hepcidin levels and hepcidin/ferritin ratios were markedly lower in the transfusion-dependent patients with ring sideroblasts. Despite the excess of iron due to RBCT, hepcidin levels were lower than expected, thereby increasing the iron uptake from the gut and release of iron from the reticulo-endothelial system. Transfusion dependency is a known risk factor for iron toxicity. However, ineffective erythropoiesis in RS subgroups evidently leads to additional iron toxicity and potentially to increased morbidity and mortality [13–15]. Therefore, transfusion-dependent LRMDS patients with ring sideroblasts should be closely monitored for signs of iron toxicity and treated accordingly.

Our data suggest that LPI levels above the LLOD are associated with inferior overall and progression-free survival, irrespective of transfusion status. This highlights the importance of rational RBCT strategies in LRMDS patients. Novel hepcidin regulators as erythroferrone, hepcidin agonists, and early start of iron chelation are subjects for future research.

Overall MDA levels, as a marker of oxidative stress, increased significantly over time in our patient group. Oxidative stress due to iron toxicity could lead to organ damage as well as mutagenesis and clonal instability contributing to a higher progression risk [9–12]. Nevertheless, MDA is not an exclusive marker for oxidative stress, future research should focus on both oxidant and antioxidant factors thereby unraveling the exact relation between iron toxicity and oxidative stress.

In conclusion, iron toxicity is associated with inferior survival in LRMDS patients. More restrictive RBCT strategies and pre-emptive iron reducing interventions may prevent or reverse these unwanted effects.

## Supplementary information

Supplemental material
