# Supplementary material for: Toxic iron species in lower-risk myelodysplastic syndrome patients: course of disease and effects on outcome
Source: Leukemia. 2020 Sep 18;35(6):1745–50. doi: 10.1038/s41375-020-01022-2 (PMC8179850; doi:10.1038/s41375-020-01022-2)
Supplement: Supplementary file 1 — Supplemental material [file 41375_2020_1022_MOESM1_ESM.docx]

**Supplemental methods**

*Biochemical analysis and reference ranges*

NTBI

Analysis of serum NTBI was based on the chelation-ultrafiltration-detection approach. NTBI is mobilized in the serum by weak iron-mobilizing chelators. The chelated NTBI is separated from transferrin-bound iron by ultrafiltration and detected by colorimetry (Zhang et al., Biochem Mol Biol, 1995). The lower limit of detection (LLOD) of the assay is 0.47 µmol/L. The reference range as assessed in 33 healthy volunteers is <0.47 to 1.98 µmol/L).

LPI

The LPI analysis was based on the measurement of the redox-active and rapidly chelatable fraction of NTBI. The assay measures iron catalyzed radical generation in the presence of a low ascorbate concentration. Fluorogenic redox sensitive probe dihydrorhodamine-123 was used to measure radical generation; iron-catalyzed radical generation was calculated by subtracting the radical generation in the presence of 50 µmol/L of the iron chelator deferiprone (Esposito et al., Blood, 2003). The LLOD of the assay is 0.24 µmol/L. The reference range as assessed in 33 healthy volunteers is <0.24 to 1.00 µmol/L. We measured LPI in 50 individuals (≥60 years) with and without anemia included in the Lifelines cohort in order to evaluate the upper limit of the reference range of our assay. Lifelines is a multi-disciplinary prospective population-based cohort study examining in a unique three-generation design the health and health-related behaviours of 167 729 persons living in the North of The Netherlands. It employs a broad range of investigative procedures in assessing the biomedical, socio-demographic, behavioural, physical and psychological factors which contribute to the health and disease of the general population, with a special focus on multi-morbidity and complex genetics (Stolk et al., Eur J Epidemiol, 2008). The LPI level was <0.24 µmol/L in all 50 age-matched elderly individuals included in the Lifelines cohort, irrespective of gender, age, and presence of anemia.

Hepcidin

The hepcidin-25 assay is based on a combination of weak cation exchange chromatography and time-of-flight mass spectrometry, using a hepcidin analogue as internal standard (Kroot et al, Clin Chem, 2010. Median reference values in a Dutch reference population were 4.5 nmol/L for men, 2.0 nmol/L for premenopausal women, and 4.9 nmol/L for postmenopausal women. (www.hepcidinanalysis.com), accessed on May 1^st^ 2018.

GDF15

GDF15 levels were analyzed by using a DuoSet (R&D Systems, Minneapolis, MN) enzyme-linked immunosorbent assay for human GDF15 following the manufacturer’s protocol.

sTfR

Serum sTfR was measured immunonephelometrically by using polystyrene particles coated with a monoclonal antibody specific to human sTfR on a BN II System (Dade Behring Marburg GmbH, Marburg, Germany).

MDA

Malondialdehyde (MDA) was measured in duplicate by spectrofluorometry (Conti et al., Clin Chem, 1991). Malondialdehyde (MDA) was measured in lithium heparin anti-coagulated plasma, or standard solution was added to 1mL reagens consisting of 10 mmol/L 2-thiobarbituric acid in phosphate buffer (0.1 mol/L, pH3). The solution was vortexed and incubated for one hour at 96^0^C. Thereafter, samples were placed on ice for 5minutes after which 2 mL of butanol was added. The mixture was shaken twice for one minute to extract TBA-MDA adduct and then centrifuged at 1500x g for 5 minutes at 4^0^C. For fluorometric measurement of the supernatant a Shimadzu RFF500 spectrofluorometer was used. The results were quantified by comparison with the standard curve, obtained with 1,1,3,3-tetramethoxy-propane, ranging from 0-10 µmol/L. The CV for this assay was <10% for both intra- and interassay variations. (Conti 1991).The LLOD of the assay is 0.12 µmol/L. The reference range of serum MDA as measured in an adult Dutch population is 0.16 to 0.64 µmol/L. Reference values in an elderly population are lacking. Therefore, MDA was measured in an age-matched reference population (≥60 years) with and without anemia in the Lifelines cohort (n=319). The LLOD of this assay was 0.22 µmol/L.

The MDA levels of anemic individuals were: median 0.78 µmol/L, mean 0.92 µmol/L (range 0.22 to 4.50); for non-anemic individuals MDA levels were: median 0.91 µmol/L, mean 0.99 µmol/L (range 0.22 to 3.10). Mean MDA levels tended to increase with age, with a mean MDA level at 65 years of 0.88 µmol/L that increased to 1.06 µmol/L at the age of 85 years. The MDA reference range for the non-anemic group (+/-2 SD) was 0.22 to 2.33 µmol/L. For this manuscript we used the reference ranges obtained from the non-anemic group.

**Supplemental figures**

*Figure S1* Scatter plot of labile plasma iron and transferrin saturation

*
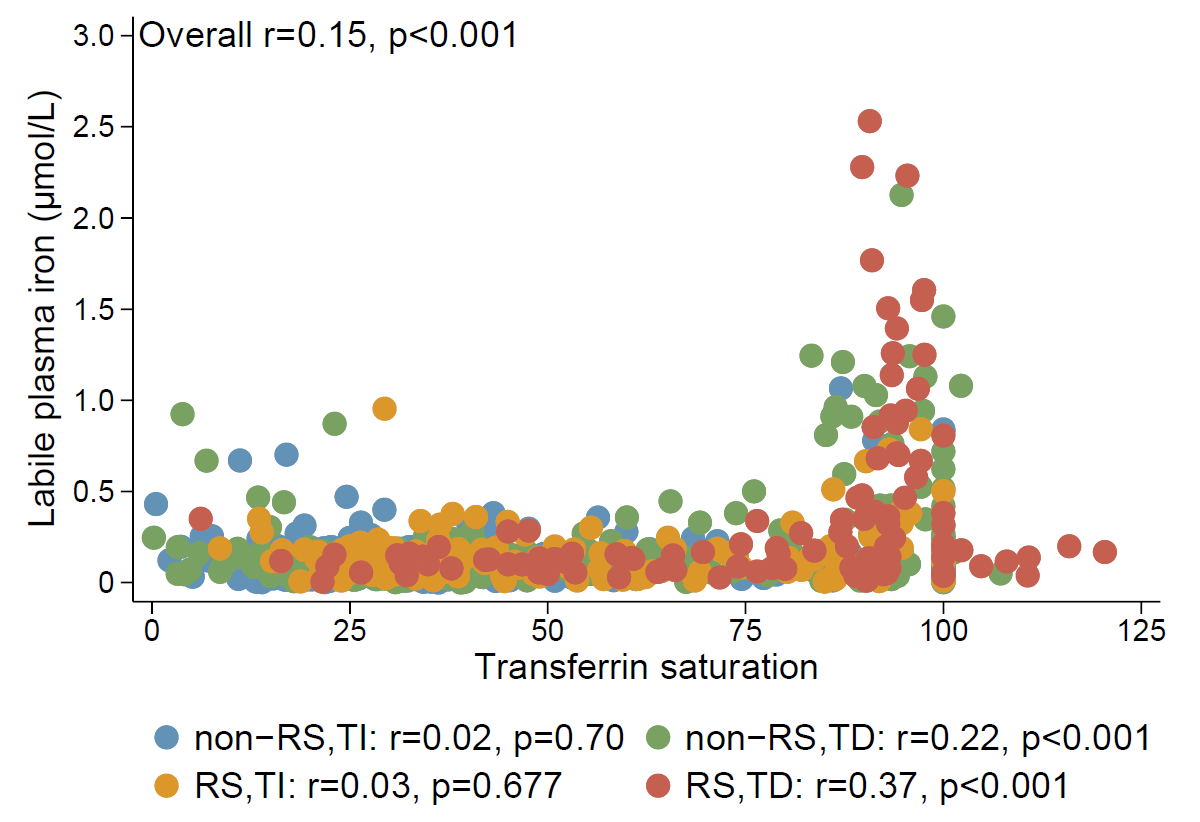
*RS: ring sideroblast; TI: transfusion-independent; TD: transfusion-dependent

*Figure S2 Scatter plot of GDF-15 and hepcidin*


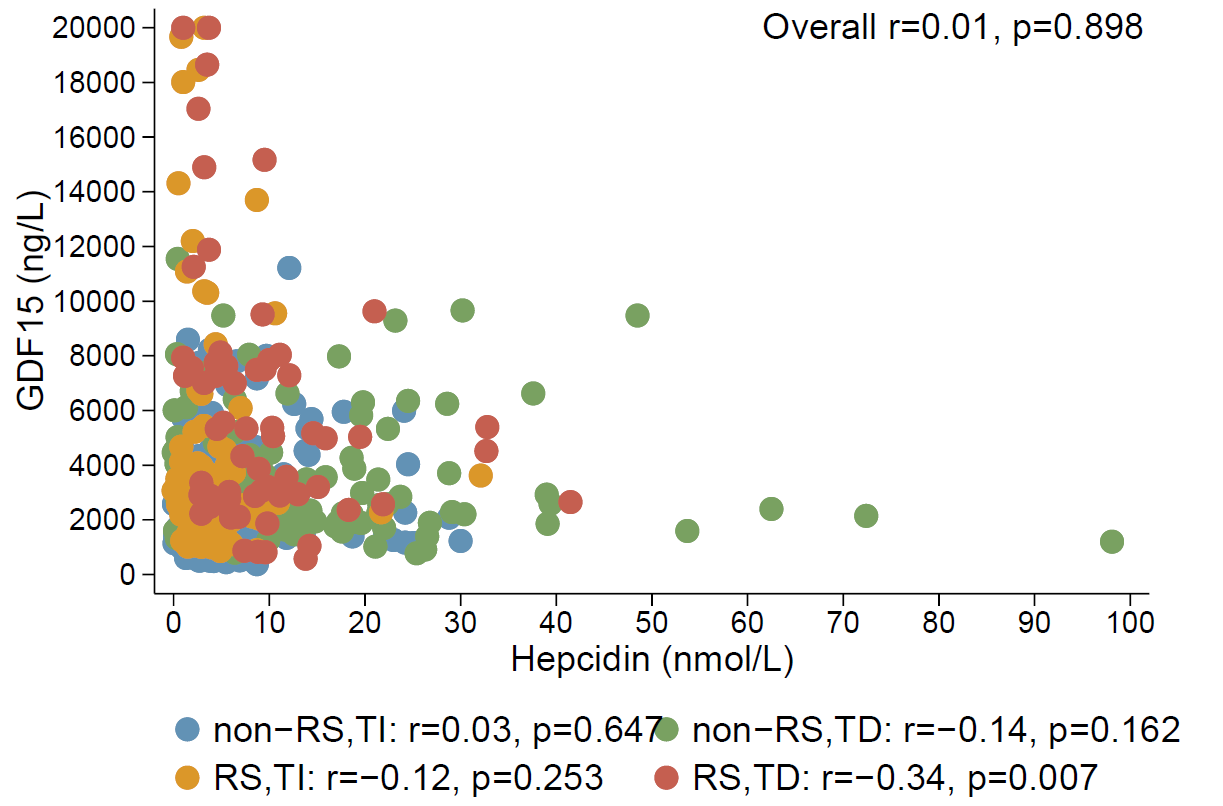
GDF15: growth differentiation factor 15; RS: ring sideroblast; TI: transfusion-independent; TD: transfusion-dependent

**Supplemental tables**

*Table S1 Iron sub study parameters for the first five visits*

|  | **Visit** | | | | | | | | | |
| --- | --- | --- | --- | --- | --- | --- | --- | --- | --- | --- |
|  | **1** | | **2** | | **3** | | **4** | | **5** | |
|  | **N** | **Mean (sd)** | **N** | **Mean (sd)** | **N** | **Mean (sd)** | **N** | **Mean (sd)** | **N** | **Mean (sd)** |
|  |  |  |  |  |  |  |  |  |  |  |
| **Total patients** | 256 |  | 251 |  | 222 |  | 176 |  | 135 |  |
|  |  |  |  |  |  |  |  |  |  |  |
| **Ferritin (µg/L)** | 252 | 488 (588) | 213 | 605 (747) | 183 | 702 (1057) | 150 | 822 (1093) | 115 | 858 (1275) |
| NonRS-TI | 140 | 280 (318) | 99 | 274 (308) | 86 | 251 (256) | 60 | 262 (371) | 51 | 263 (223) |
| NonRS-TD | 46 | 935 (863) | 55 | 1145 (1070) | 53 | 1297 (1510) | 47 | 1083 (1128) | 32 | 1399 (1519) |
| RS-TI | 48 | 505 (567) | 35 | 356 (283) | 26 | 343 (244) | 21 | 493 (416) | 16 | 441 (314) |
| RS-TD | 18 | 919 (559) | 24 | 1096 (650) | 18 | 1627 (1151) | 22 | 2104 (1505) | 16 | 2092 (1918) |
| Ref. range:  20-300 µg/L |  |  |  |  |  |  |  |  |  |  |
| **TSAT (%)** | 254 | 50 (29) | 217 | 50 (29) | 191 | 49 (31) | 158 | 52 (31) | 120 | 54 (31) |
| NonRS-TI | 143 | 44 (29) | 100 | 43 (28) | 91 | 43 (29) | 66 | 46 (31) | 52 | 41 (28) |
| NonRS-TD | 46 | 57 (28) | 57 | 54 (30) | 55 | 55 (33) | 48 | 49 (31) | 34 | 56 (32) |
| RS-TI | 48 | 53 (26) | 36 | 52 (27) | 26 | 41 (20) | 22 | 56 (29) | 17 | 57 (27) |
| RS-TD | 17 | 70 (29) | 24 | 71 (26) | 19 | 73 (33) | 22 | 76 (23) | 17 | 88 (15) |
| Ref. range:  15-45% |  |  |  |  |  |  |  |  |  |  |
| **LPI (µmol/L)** | 255 | 0.17 (0.25) | 216 | 0.18 (0.45) | 187 | 0.17 (0.22) | 152 | 0.17 (0.23) | 116 | 0.22 (0.35) |
| NonRS-TI | 143 | 0.14 (0.12) | 100 | 0.11 (0.12) | 88 | 0.11 (0.06) | 62 | 0.11 (0.11) | 52 | 0.12 (0.08) |
| NonRS-TD | 46 | 0.19 (0.33) | 57 | 0.30 (0.77) | 54 | 0.21 (0.31) | 48 | 0.18 (0.17) | 32 | 0.25 (0.33) |
| RS-TI | 48 | 0.16 (0.18) | 35 | 0.15 (0.15) | 26 | 0.15 (0.11) | 21 | 0.16 (0.16) | 16 | 0.11 (0.07) |
| RS-TD | 18 | 0.41 (0.61) | 24 | 0.27 (0.50) | 19 | 0.36 (0.37) | 21 | 0.36 (0.47) | 16 | 0.59 (0.69) |
| Ref. value:  <0.24 µmol/L |  |  |  |  |  |  |  |  |  |  |
| **NTBI (µmol/L)** | 254 | 1.03 (1.28) | 216 | 1.15 (1.41) | 188 | 1.18 (1.53) | 152 | 1.18 (1.46) | 116 | 1.50 (1.78) |
| NonRS-TI | 141 | 0.64 (0.69) | 100 | 0.72 (0.73) | 88 | 0.58 (0.52) | 62 | 0.53 (0.60) | 52 | 0.69 (0.76) |
| NonRS-TD | 47 | 1.44 (1.62) | 56 | 1.50 (1.84) | 55 | 1.49 (1.53) | 48 | 1.28 (1.55) | 32 | 1.85 (2.06) |
| RS-TI | 48 | 1.20 (1.33) | 36 | 1.15 (1.30) | 26 | 1.44 (1.97) | 21 | 1.67 (1.86) | 16 | 1.82 (1.86) |
| RS-TD | 18 | 2.51 (2.09) | 24 | 2.15 (1.89) | 19 | 2.69 (2.44) | 21 | 2.36 (1.68) | 16 | 3.09 (2.15) |
| Ref. range:  <0.47-1.98 µmol/L) |  |  |  |  |  |  |  |  |  |  |
| **hepcidin** | 246 | 8.6 (12.0) | 206 | 9.4 (11.4) | 188 | 10.2 (13.4) | 154 | 10.2 (12.1) | 116 | 8.9 (11.1) |
| NonRS-TI | 140 | 6.2 (6.2) | 98 | 6.4 (6.7) | 89 | 5.6 (4.8) | 62 | 6.5 (6.8) | 52 | 5.2 (5.0) |
| NonRS-TD | 44 | 20.4 (21.7) | 53 | 18.8 (16.6) | 55 | 21.0 (19.3) | 48 | 15.7 (14.2) | 32 | 16.0 (13.5) |
| RS-TI | 44 | 5.0 (5.8) | 32 | 3.9 (3.5) | 26 | 3.7 (2.8) | 22 | 3.8 (3.7) | 16 | 3.9 (3.5) |
| RS-TD | 18 | 7.8 (5.2) | 23 | 8.4 (6.6) | 18 | 9.3 (10.0) | 22 | 15.0 (16.9) | 16 | 11.7 (17.0) |
| Ref. range:  ♂ <0.5-14.7  ♀*0.2-15.6 nmol/L |  |  |  |  |  |  |  |  |  |  |
| **GDF-15** | 99 | 3051 (3034) | 92 | 3309 (2892) | 76 | 3372 (2993) | 76 | 4216 (4058) | 62 | 3516 (2935) |
| NonRS-TI | 53 | 2428 (1846) | 40 | 2318 (1726) | 32 | 2382 (2053) | 24 | 2756 (2842) | 25 | 2468 (2008) |
| NonRS-TD | 13 | 2467 (1365) | 20 | 3488 (2149) | 20 | 3644 (2525) | 26 | 4176 (2412) | 18 | 3620 (2283) |
| RS-TI | 26 | 4578 (4917) | 19 | 3113 (2678) | 16 | 4083 (3714) | 13 | 4129 (4449) | 8 | 3885 (2860) |
| RS-TD | 7 | 3187 (1418) | 13 | 6369 (4706) | 8 | 5226 (4546) | 13 | 7082 (6508) | 11 | 5460 (4613) |
| Ref. range:  7.8-500 ng/l |  |  |  |  |  |  |  |  |  |  |
| **sTfR** | 99 | 1.5 (0.9) | 92 | 1.7 (1.1) | 77 | 1.7 (1.2) | 76 | 2.0 (2.7) | 61 | 1.6 (0.9) |
| NonRS-TI | 53 | 1.5 (0.9) | 40 | 1.7 (1.2) | 32 | 1.8 (1.5) | 24 | 1.6 (0.8) | 25 | 1.5 (0.7) |
| NonRS-TD | 13 | 1.3 (0.8) | 20 | 1.4 (1.2) | 21 | 1.2 (0.9) | 26 | 2.4 (4.5) | 17 | 1.5 (1.2) |
| RS-TI | 26 | 1.8 (0.8) | 19 | 1.9 (0.8) | 16 | 2.0 (0.7) | 13 | 2.3 (0.8) | 8 | 2.0 (0.7) |
| RS-TD | 7 | 1.3 (1.1) | 13 | 1.9 (1.2) | 8 | 1.6 (0.9) | 13 | 1.6 (1.2) | 11 | 1.6 (1.2) |
| Ref. range:  0.76-1.76 mg/l |  |  |  |  |  |  |  |  |  |  |
| **MDA** | 121 | 3.0 (11.3) | 101 | 2.0 (3.8) | 74 | 1.4 (1.8) | 68 | 2.1 (3.4) | 48 | 3.9 (7.1) |
| NonRS-TI | 45 | 2.2 (3.7) | 31 | 1.8 (2.4) | 22 | 2.1 (2.7) | 14 | 1.5 (1.6) | 13 | 1.4 (0.8) |
| NonRS-TD | 31 | 1.5 (1.8) | 32 | 1.3 (0.9) | 25 | 0.9 (0.5) | 23 | 1.6 (3.2) | 12 | 2.6 (2.3) |
| RS-TI | 34 | 6.2 (20.6) | 22 | 2.0 (3.4) | 16 | 1.0 (0.5) | 18 | 3.0 (4.7) | 12 | 3.5 (7.9) |
| Ref. range:  0.22-3.10 µmol/l |  |  |  |  |  |  |  |  |  |  |
| **Hepcidin/Ferritin ratio** | 242 | 0.025 (0.022) | 203 | 0.024 (0.023) | 183 | 0.042 (0.208) | 150 | 0.026 (0.032) | 115 | 0.023 (0.028) |
| NonRS-TI | 137 | 0.030 (0.025) | 98 | 0.032 (0.029) | 86 | 0.070 (0.301) | 60 | 0.041 (0.041) | 51 | 0.033 (0.037) |
| NonRS-TD | 43 | 0.023 (0.013) | 51 | 0.020 (0.016) | 53 | 0.022 (0.018) | 47 | 0.021 (0.023) | 32 | 0.020 (0.018) |
| RS-TI | 44 | 0.014 (0.013) | 31 | 0.013 (0.011) | 26 | 0.014 (0.012) | 21 | 0.012 (0.010) | 16 | 0.013 (0.014) |
| RS-TD  Ref. range:  ♂0.003-0.088  ♀*0.009-0,143 pmol/µg | 18 | 0.011 (0.013) | 23 | 0.011 (0.010) | 18 | 0.007 (0.004) | 22 | 0.009 (0.008) | 16 | 0.007 (0.007) |

sd: standard deviation; RS: ring sideroblasts; TI: transfusion-independent: TD: transfusion-dependent; TSAT: transferrin saturation; LPI: labile plasma iron; NTBI: non-transferrin bound iron; GDF-15: growth differentiation factor 15: sTfR: soluble transferrin receptor; MDA: malondialdehyde. *post-menopausal women.

*Table S2 Iron and oxidative stress parameters for the first five visits (median, p10 and p90) coefficients from linear quantile regression*

|  | **Visit** | | | | | | | | | | **Beta (95% CI)** | **p** |
| --- | --- | --- | --- | --- | --- | --- | --- | --- | --- | --- | --- | --- |
|  | **1** | | **2** | | **3** | | **4** | | **5** | |  |  |
|  | **N** | **Median (p10-p90)** | **N** | **Median (p10-p90)** | **N** | **Median (p10-p90)** | **N** | **Median (p10-p90)** | **N** | **Median (p10-p90)** |  |  |
|  |  |  |  |  |  |  |  |  |  |  |  |  |
| **Total patients** | 256 |  | 251 |  | 222 |  | 176 |  | 135 |  |  |  |
|  |  |  |  |  |  |  |  |  |  |  |  |  |
| **Ferritin (µg/L)** | 252 | 305 (58 - 1078) | 213 | 357 (69 - 1427) | 183 | 293 (61 - 1794) | 150 | 349 (69 - 2322) | 115 | 374 (61 - 2560) | 122.03 (99.1 - 144.96) | <0.001 |
| Non-RS, TI | 140 | 188 (47 - 577) | 99 | 202 (40 - 541) | 86 | 177 (36 - 584) | 60 | 181 (45 - 471) | 51 | 206 (51 - 603) | -2.83 (-494.73 - 489.08) | 0.99 |
| Non-RS, TD | 46 | 673 (106 - 1982) | 55 | 883 (109 - 2432) | 53 | 809 (97 - 2554) | 47 | 673 (120 - 2372) | 32 | 858 (180 - 3169) | 328.88 (-992.37 - 1650.13) | 0.63 |
| RS, TI | 48 | 326 (123 - 1108) | 35 | 271 (91 - 751) | 26 | 275 (107 - 796) | 21 | 388 (175 - 999) | 16 | 359 (191 - 818) | -8.27 (-95.09 - 78.55) | 0.85 |
| RS, TD | 18 | 889 (108 - 1382) | 24 | 1116 (230 - 1856) | 18 | 1439 (518 - 2622) | 22 | 2081 (223 - 3560) | 16 | 1178 (474 - 4657) | 454.46 (334.65 - 574.27) | <0.001 |
|  |  |  |  |  |  |  |  |  |  |  |  |  |
| **TSAT (%)** | 254 | 39 (19 - 100) | 217 | 41 (19 - 97) | 191 | 37 (16 - 100) | 158 | 40 (19 - 100) | 120 | 42 (21 - 100) | 1.5 (0.26 - 2.74) | 0.02 |
| Non-RS, TI | 143 | 34 (18 - 100) | 100 | 33 (18 - 100) | 91 | 33 (17 - 100) | 66 | 34 (17 - 100) | 52 | 30 (17 - 100) | 0.28 (-1.12 - 1.68) | 0.7 |
| Non-RS, TD | 46 | 51 (26 - 100) | 57 | 54 (16 - 96) | 55 | 42 (14 - 100) | 48 | 37 (16 - 100) | 34 | 53 (20 - 100) | 2.47 (0.46 - 4.47) | 0.02 |
| RS, TI | 48 | 44 (24 - 95) | 36 | 46 (24 - 95) | 26 | 37 (22 - 73) | 22 | 56 (22 - 92) | 17 | 51 (26 - 93) | 1.31 (-0.24 - 2.87) | 0.1 |
| RS, TD | 17 | 84 (31 - 94) | 24 | 78 (31 - 94) | 19 | 90 (16 - 102) | 22 | 81 (45 - 100) | 17 | 93 (59 - 100) | 3.66 (0.91 - 6.4) | 0.01 |
|  |  |  |  |  |  |  |  |  |  |  |  |  |
| **LPI (µMol/L)** | 255 | 0.12 (0.03 - 0.31) | 216 | 0.11 (0.03 - 0.27) | 187 | 0.12 (0.03 - 0.34) | 152 | 0.12 (0.02 - 0.30) | 116 | 0.12 (0.03 - 0.67) | 0.01 (0 - 0.02) | 0.01 |
| Non-RS, TI | 143 | 0.12 (0.02 - 0.20) | 100 | 0.08 (0.02 - 0.19) | 88 | 0.11 (0.03 - 0.19) | 62 | 0.09 (0.02 - 0.18) | 52 | 0.11 (0.04 - 0.20) | 0 (-0.01 - 0.01) | 0.84 |
| Non-RS, TD | 46 | 0.09 (0.02 - 0.34) | 57 | 0.12 (0.04 - 0.76) | 54 | 0.16 (0.04 - 0.36) | 48 | 0.15 (0.02 - 0.44) | 32 | 0.13 (0.03 - 0.88) | 0.02 (-0.02 - 0.06) | 0.33 |
| RS, TI | 48 | 0.12 (0.02 - 0.33) | 35 | 0.13 (0.02 - 0.25) | 26 | 0.13 (0.03 - 0.34) | 21 | 0.11 (0.03 - 0.30) | 16 | 0.10 (0.01 - 0.20) | 0 (-0.01 - 0.01) | 0.81 |
| RS, TD | 18 | 0.17 (0.08 - 1.77) | 24 | 0.13 (0.06 - 0.39) | 19 | 0.18 (0.04 - 1.14) | 21 | 0.16 (0.07 - 1.25) | 16 | 0.26 (0.01 - 1.60) | 0.07 (-0.02 - 0.16) | 0.11 |
|  |  |  |  |  |  |  |  |  |  |  |  |  |
| **NTBI (µMol/L)** | 254 | 0.53 (0.10 - 2.92) | 216 | 0.57 (0.13 - 2.94) | 188 | 0.56 (0.12 - 3.33) | 152 | 0.53 (0.10 - 3.24) | 116 | 0.66 (0.15 - 4.17) | 0.08 (0.03 - 0.12) | 0 |
| Non-RS, TI | 141 | 0.44 (0.10 - 1.25) | 100 | 0.51 (0.15 - 1.50) | 88 | 0.45 (0.12 - 1.13) | 62 | 0.40 (0.06 - 0.92) | 52 | 0.49 (0.06 - 1.43) | -0.01 (-0.03 - 0.02) | 0.56 |
| Non-RS, TD | 47 | 0.79 (0.10 - 3.82) | 56 | 0.67 (0.10 - 3.85) | 55 | 0.73 (0.18 - 3.75) | 48 | 0.57 (0.08 - 4.25) | 32 | 0.93 (0.19 - 5.47) | 0.27 (0.15 - 0.39) | <0.001 |
| RS, TI | 48 | 0.66 (0.11 - 3.85) | 36 | 0.45 (0.07 - 3.33) | 26 | 0.58 (0.07 - 3.52) | 21 | 0.90 (0.14 - 4.15) | 16 | 1.00 (0.26 - 4.92) | 0.09 (-0.03 - 0.2) | 0.14 |
| RS, TD | 18 | 2.46 (0.33 - 5.39) | 24 | 1.92 (0.25 - 4.25) | 19 | 3.20 (0.06 - 5.20) | 21 | 2.52 (0.26 - 4.93) | 16 | 2.85 (0.42 - 6.90) | 0.22 (0.01 - 0.42) | 0.04 |
|  |  |  |  |  |  |  |  |  |  |  |  |  |
| **Hepcidin (nmol/L)** | 246 | 4.7 (0.8 - 21.7) | 206 | 5.2 (0.7 - 24.2) | 188 | 5.6 (0.5 - 24.5) | 154 | 5.7 (1.3 - 25.8) | 116 | 4.7 (0.8 - 22.4) | 0.57 (0.34 - 0.79) | <0.001 |
| Non-RS, TI | 140 | 4.2 (0.8 - 14.3) | 98 | 4.3 (0.5 - 14.8) | 89 | 4.4 (0.5 - 13.8) | 62 | 4.0 (1.6 - 13.1) | 52 | 3.4 (0.8 - 10.8) | -0.02 (-0.38 - 0.33) | 0.89 |
| Non-RS, TD | 44 | 13.2 (1.1 - 51.0) | 53 | 18.1 (1.2 - 39.1) | 55 | 17.7 (0.4 - 41.8) | 48 | 12.2 (0.4 - 37.9) | 32 | 12.6 (1.3 - 32.3) | 0.86 (-0.62 - 2.35) | 0.26 |
| RS, TI | 44 | 3.4 (0.8 - 9.2) | 32 | 2.8 (0.5 - 8.2) | 26 | 3.1 (0.5 - 7.5) | 22 | 2.5 (1.1 - 8.9) | 16 | 3.1 (0.7 - 10.6) | -0.04 (-0.36 - 0.29) | 0.82 |
| RS, TD | 18 | 7.8 (1.5 - 15.9) | 23 | 7.2 (2.1 - 19.5) | 18 | 8.9 (1.5 - 19.5) | 22 | 10.7 (1.3 - 37.6) | 16 | 4.2 (1.0 - 41.5) | 1.67 (0.52 - 2.82) | 0 |
|  |  |  |  |  |  |  |  |  |  |  |  |  |
| **GDF-15 (ng/L)** | 99 | 2189 (938 - 5956) | 92 | 2491 (1000 - 7290) | 76 | 2470 (1016 - 7982) | 76 | 2932 (887 - 8058) | 62 | 2556 (1045 - 7488) | 229.08 (133.23 - 324.93) | <0.001 |
| Non-RS, TI | 53 | 1831 (731 - 4658) | 40 | 1735 (721 - 4596) | 32 | 1568 (615 - 5684) | 24 | 1520 (574 - 7615) | 25 | 1667 (633 - 5736) | 118.87 (-68.36 - 306.09) | 0.21 |
| Non-RS, TD | 13 | 1856 (1204 - 4828) | 20 | 2971 (1067 - 6895) | 20 | 2583 (1844 - 7166) | 26 | 3494 (1609 - 8058) | 18 | 3064 (1398 - 8037) | 523.54 (254.19 - 792.89) | <0.001 |
| RS, TI | 26 | 2774 (996 - 11083) | 19 | 2402 (1067 - 7354) | 16 | 2694 (1223 - 10303) | 13 | 3099 (1474 - 5435) | 8 | 2780 (1331 - 9554) | 225.25 (-35.96 - 486.46) | 0.09 |
| RS, TD | 7 | 2883 (1869 - 5370) | 13 | 5035 (2571 - 11253) | 8 | 3538 (830 - 15167) | 13 | 4517 (869 - 20000) | 11 | 5166 (1053 - 7933) | 363.24 (-45.59 - 772.07) | 0.08 |
|  |  |  |  |  |  |  |  |  |  |  |  |  |
| **sTfR (mg/L)** | 99 | 1.3 (0.7 - 2.8) | 92 | 1.3 (0.7 - 3.1) | 77 | 1.4 (0.7 - 3.0) | 76 | 1.4 (0.7 - 3.4) | 61 | 1.3 (0.8 - 2.7) | 0.03 (-0.03 - 0.09) | 0.38 |
| Non-RS, TI | 53 | 1.2 (0.8 - 2.7) | 40 | 1.3 (0.8 - 2.9) | 32 | 1.4 (0.9 - 3.0) | 24 | 1.4 (0.9 - 2.8) | 25 | 1.2 (0.9 - 2.7) | 0.02 (-0.02 - 0.05) | 0.32 |
| Non-RS, TD | 13 | 1.1 (0.6 - 2.5) | 20 | 1.0 (0.5 - 2.8) | 21 | 1.1 (0.4 - 1.8) | 26 | 1.2 (0.4 - 3.6) | 17 | 1.2 (0.6 - 2.2) | 0 (-0.69 - 0.69) | 1 |
| RS, TI | 26 | 1.6 (0.9 - 3.3) | 19 | 1.8 (1.0 - 3.1) | 16 | 2.0 (1.1 - 2.8) | 13 | 2.2 (1.3 - 3.5) | 8 | 2.2 (1.0 - 2.8) | 0.11 (0.07 - 0.15) | <0.001 |
| RS, TD | 7 | 0.9 (0.4 - 3.1) | 13 | 1.9 (0.6 - 3.4) | 8 | 1.4 (0.6 - 3.1) | 13 | 1.4 (0.5 - 3.2) | 11 | 1.4 (0.4 - 3.6) | -0.12 (-0.2 - -0.04) | 0.01 |
|  |  |  |  |  |  |  |  |  |  |  |  |  |
| **MDA (µMol/L)** | 121 | 1.0 (0.5 - 4.1) | 101 | 1.1 (0.5 - 3.9) | 74 | 0.9 (0.4 - 2.0) | 68 | 1.0 (0.5 - 6.7) | 48 | 1.2 (0.7 - 11.9) | 0 (-47.8 - 47.8) | 1 |
| Non-RS, TI | 45 | 1.1 (0.5 - 3.7) | 31 | 0.9 (0.4 - 3.9) | 22 | 1.1 (0.4 - 4.6) | 14 | 0.9 (0.5 - 2.4) | 13 | 1.1 (0.6 - 2.6) | -0.03 (-0.31 - 0.25) | 0.84 |
| Non-RS, TD | 31 | 1.0 (0.4 - 2.8) | 32 | 1.1 (0.7 - 1.6) | 25 | 0.8 (0.1 - 1.7) | 23 | 0.8 (0.3 - 1.9) | 12 | 1.6 (0.8 - 6.4) | -0.17 (-0.41 - 0.07) | 0.17 |
| RS, TI | 34 | 1.1 (0.6 - 10.2) | 22 | 0.9 (0.4 - 4.3) | 16 | 0.9 (0.4 - 1.4) | 18 | 0.9 (0.5 - 12.8) | 12 | 1.1 (0.5 - 3.8) | -0.88 (-14.4 - 12.65) | 0.9 |
| RS, TD | 11 | 1.1 (0.6 - 1.4) | 16 | 1.1 (0.4 - 5.6) | 11 | 0.9 (0.5 - 4.1) | 13 | 1.4 (0.5 - 7.6) | 11 | 1.9 (0.7 - 28.5) | 0.5 (-1.03 - 2.03) | 0.52 |

Beta: coefficient indicating the median value change per visit; CI: confidence interval; RS: ring sideroblasts; TI: transfusion-independent: TD: transfusion-dependent; TSAT: transferrin saturation; LPI: labile plasma iron; NTBI: non-transferrin bound iron; GDF-15: growth differentiation factor 15: sTfR: soluble transferrin receptor; MDA: malondialdehyde.

*Table S3.1 MDA by MDS diagnosis at first visit*

|  | **N** | **Mean (sd)** | **Median (min - max)** |
| --- | --- | --- | --- |
| Total | 121 | 3.0 (11.3) | 1.0 (0.0 - 120.0) |
|  |  |  |  |
| RA | 17 | 3.8 (5.5) | 1.3 (0.0 - 18.0) |
| RARS | 38 | 2.5 (4.3) | 1.1 (0.5 - 23.0) |
| RCMD | 51 | 1.2 (1.0) | 0.9 (0.0 - 4.7) |
| RCMD-RS | 7 | 18.3 (44.8) | 1.3 (0.6 - 120.0) |
| RAEB-1 | 4 | 1.0 (0.5) | 0.9 (0.6 - 1.7) |
| 5q-Syndrome | 4 | 4.1 (4.6) | 2.2 (1.1 - 10.9) |

MDA: malondialdehyde; MDS myelodysplastic syndrome; sd: standard deviation; min: minimum; max: maximum; RA: refractory anemia; RARS: refractory anemia with ring sideroblasts; RCMD: refractory cytopenia with multilineage dysplasia; RCMD-RS: refractory cytopenia with multilineage dysplasia and ring sideroblasts; RAEB: refractory anemia with excess of blasts; 5q-: deletion of the long arm of chromosome 5

*Table S3.2 Baseline characteristics comparing patients with MDA levels below or above the median**

|  | MDA below median (<=1µmol/L) | MDA above median (>1µmol/L) |
| --- | --- | --- |
| **Number of patients** | 61 | 60 |
|  |  |  |
| **Mean age (sd), years**** | 74.2 (8.6) | 70.0 (11.1) |
|  |  | p=0.02 |
| **Sex N (%), males**** | 45 (73.8) | 31 (51.7) |
|  |  | p=0.012 |
| **MDS diagnosis:** |  |  |
| RA | 4 (6.6) | 13 (21.7) |
| RARS | 19 (31.1) | 19 (31.7) |
| RCMD | 32 (52.5) | 19 (31.7) |
| RCMD-RS | 3 (4.9) | 4 (6.7) |
| RAEB-1 | 3 (4.9) | 1 (1.7) |
| 5q-Syndrome | 0 (0.0) | 4 (6.7) |
|  |  | p=0.021 |
| **Group:** |  |  |
| nonRS-TI | 22 (36.1) | 23 (38.3) |
| NonRS-TD | 17 (27.9) | 14 (23.3) |
| RS-TI | 17 (27.9) | 17 (28.3) |
| RS-TD | 5 (8.2) | 6 (10.0) |
|  |  | p=0.94 |
| **Transfusion density:** |  |  |
| Mean (sd) | 0.54 (1.28) | 1.14 (4.25) |
| Median (min-max) | 0.00 (0.00 - 8.30) | 0.00 (0.00 - 30.44) |
| 0 | 45 (73.8) | 41 (68.3) |
| >0 - <0.75 | 1 (1.6) | 3 (5.0) |
| ≥0.75 - ≤1.75 | 8 (13.1) | 10 (16.7) |
| >1.75 | 7 (11.5) | 6 (10.0) |
|  |  | p=0.688 |
| **IPSSR category:** |  |  |
| very low/low | 45 (73.8) | 47 (78.3) |
| Intermediate | 8 (13.1) | 2 (3.3) |
| high/very high | 3 (4.9) | 1 (1.7) |
| Unknown | 5 (8.2) | 10 (16.7) |
|  |  | p=0.098 |
| **IPSS category:** |  |  |
| Low risk | 7 (11.5) | 11 (18.3) |
| Intermed-1 | 31 (50.8) | 31 (51.7) |
| Intermed-2 | 22 (36.1) | 18 (30.0) |
| Unknown | 1 (1.6) | 0 (0.0) |
|  |  | p=0.516 |
| **Karnofsky performance status:** |  |  |
| Able to work and normal activity | 3 (4.9) | 2 (3.3) |
| Unable to work | 0 (0.0) | 0 (0.0) |
| Unable to care for self | 20 (32.8) | 9 (15.0) |
| Unknown | 38 (62.3) | 49 (81.7) |
|  |  | p=0.060 |
| **Comorbidity index:** |  |  |
| Low risk | 34 (55.7) | 37 (61.7) |
| Intermediate risk | 22 (36.1) | 20 (33.3) |
| High risk | 5 (8.2) | 3 (5.0) |
|  |  | p=0.700 |
| **EQ-5D index score (N):** | 51 | 31 |
| Mean (sd) | 0.73 (0.29) | 0.80 (0.18) |
| Median (p10-p90) | 0.80 (0.33 - 1.00) | 0.77 (0.62 - 1.00) |
|  |  | p=0.28 |
| **EQ-5D VAS score (N):** | 58 | 56 |
| Mean (sd) | 68.88 (20.92) | 74.84 (16.83) |
| Median (p10-p90) | 75.00 (30.00 - 90.00) | 75.00 (50.00 - 100.00) |
|  |  | p=0.0972 |
|  |  |  |
| **ESA use during observation period (%):** | 21 (34.4) | 26 (43.3) |
|  |  | p=0.315 |
|  |  |  |
| **Iron chelation use during observation period (%):** | 5 (8.2) | 5 (8.3) |
|  |  | p=0.978 |
|  |  |  |
| **Hypomethylating agent use during observation period (%):** | 2 (1.9) | 0 (0.0) |
|  |  | p=0.644 |
|  |  |  |
| **Overall survival Median (95% CI):** | 4.0 (3.1 - 7.1) | 7.1 (5.1 - .) |
|  |  |  |
| **Cause of death:** | 35 (100.0) | 27 (100.0) |
| MDS unrelated | 10 (28.6) | 7 (25.9) |
| MDS related | 17 (48.6) | 13 (48.1) |
| Unknown | 8 (22.9) | 7 (25.9) |
|  |  | p=0.952 |

*at first visit

MDA: malondialdehyde; RA: refractory anemia; RARS: refractory anemia with ring sideroblasts; RCMD: refractory cytopenia with multilineage dysplasia; RCMD-RS: refractory cytopenia with multilineage dysplasia with ring sideroblasts; RAEB: refractory anemia with excess blasts; RS: ring sideroblasts; TI: transfusion-independent; TD: transfusion-dependent; sd: standard deviation; IPSS(-R): (revised) international prognostic scoring system; EQ5D: EuroQoL five dimension scale; ESA: erythroid stimulating agent; CI: confidence interval.

**MDA levels above the median were more frequently observed in relative young males. Currently, the etiology is not yet elucidated.

Table S3.3 MDA at baseline and highest available measurement after baseline by ring sideroblast and transfusion status

|  | MDA at baseline | | | Highest MDA level in follow-up samples | | |  | P^1^ |
| --- | --- | --- | --- | --- | --- | --- | --- | --- |
|  | N | Mean (sd) | Median (min-max) | N | Mean (sd) | Median (min-max) |  |  |
| Total | 132 | 2.8 (10.7) | 1.0 (0.0 - 120.0) | 132 | 3.2 (5.7) | 1.3 (0.1 - 32.9) |  | <0.0001 |
| nonRS-TI | 32 | 1.9 (3.2) | 1.0 (0.0 - 18.0) | 32 | 2.2 (3.0) | 1.2 (0.1 - 16.5) |  | 0.0741 |
| nonRS-TI > TD | 17 | 1.8 (2.5) | 0.9 (0.5 - 10.9) | 17 | 4.1 (7.2) | 1.5 (0.4 - 27.8) |  | 0.0351 |
| nonRS-TD | 31 | 1.6 (1.8) | 1.0 (0.3 - 9.9) | 31 | 1.4 (1.1) | 1.2 (0.1 - 5.9) |  | 0.5112 |
| RS-TI | 29 | 6.8 (22.3) | 1.0 (0.5 - 120.0) | 29 | 3.5 (6.0) | 1.3 (0.4 - 28.5) |  | 0.0727 |
| RS-TI >TD | 10 | 2.0 (1.4) | 1.7 (0.6 - 4.3) | 10 | 10.7 (12.0) | 5.6 (0.7 - 32.9) |  | 0.0284 |
| RS-TD | 13 | 1.4 (1.4) | 1.1 (0.1 - 4.9) | 13 | 2.1 (2.3) | 1.4 (0.4 - 9.0) |  | 0.0041 |

^1^ Wilcoxon matched-pairs signed-ranks test

MDA: malondialdehyde; sd: standard deviation; min: minimum; max: maximum; RS: ring sideroblast; TI: transfusion-independent; TD: transfusion-dependent; > indicates ‘evolved in’

*Table S4 Cox model of overall survival by labile plasma iron level (above or below the lower limit of detection) and transfusion status as time-varying variables*

|  | **Unadjusted** | |  | **Adjusted^1^** | |
| --- | --- | --- | --- | --- | --- |
|  | **Hazard ratio (95% CI)** | **p** |  | **Hazard ratio (95% CI)** | **p** |
| Labile plasma iron < LLOD | 1 | - |  | 1 | - |
| Labile plasma iron ≥ LLOD | 2.8 (1.5 - 5.1) | 0.001 |  | 2.7 (1.5 - 5.0) | 0.001 |
|  |  |  |  |  |  |
| Labile plasma iron < LLOD, TI | 1 | - |  | 1 | - |
| Labile plasma iron ≥ LLOD, TI | 4.1 (1.3 - 12.9) | 0.01 |  | 4.5 (1.4 - 13.9) | 0.01 |
| Labile plasma iron < LLOD, TD | 4.9 (1.9 - 12.7) | 0.001 |  | 3.9 (1.5 - 10.4) | 0.006 |
| Labile plasma iron ≥ LLOD, TD | 8.3 (3.2 - 21.4) | <0.001 |  | 6.7 (2.5 - 17.6) | <0.001 |
|  |  |  |  |  |  |

^1^adjusted for age at diagnosis and IPSS-R category

IPSS-R: revised international prognostic scoring system; CI: confidence interval; LLOD: lower limit of detection; TI: transfusion-independent; TD: transfusion-dependent

*Table S5 Cox model of overall survival by non-transferrin bound iron and transfusion status as time-varying variables*

|  | **Unadjusted** | |  | **Adjusted^1^** | |
| --- | --- | --- | --- | --- | --- |
|  | **Hazard ratio (95% CI)** | **p** |  | **Hazard ratio (95% CI)** | **p** |
| Non-transferrin bound iron normal^2^ | 1 | - |  | 1 | - |
| Non-transferrin bound iron elevated^2^ | 2.0 (1.1 - 3.6) | 0.03 |  | 1.6 (0.8 - 3.1) | 0.17 |
|  |  |  |  |  |  |
| Non-transferrin bound iron normal, TI | 1 | - |  | 1 | - |
| Non-transferrin bound iron elevated, TI | 4.6 (1.5 - 14.5) | 0.01 |  | 4.5 (1.4 - 14.4) | 0.01 |
| Non-transferrin bound iron normal, TD | 6.1 (2.6 - 14.6) | <0.001 |  | 5.2 (2.1 - 12.6) | <0.001 |
| Non-transferrin bound iron elevated, TD | 5.3 (2.1 - 13.3) | <0.001 |  | 3.7 (1.4 - 9.9) | 0.01 |
|  |  |  |  |  |  |

^1^adjusted for age at diagnosis and IPSS-R category; ^2^above or below upper limit of reference range.

CI: confidence interval; TI: transfusion-independent; TD: transfusion-dependent
